# *In utero* exposure to low dose arsenic via drinking water impairs early life lung mechanics in mice

**DOI:** 10.1186/2050-6511-14-13

**Published:** 2013-02-18

**Authors:** Kathryn A Ramsey, Alexander N Larcombe, Peter D Sly, Graeme R Zosky

**Affiliations:** 1 Division of Clinical Sciences, Telethon Institute for Child Health Research, 100 Roberts Road, Subiaco, WA, 6008, Australia; 2 Centre for Child Health Research, University of Western Australia, 100 Roberts Road, Subiaco, WA, 6008, Australia; 3 Queensland Children’s Medical Research Institute, University of Queensland, Level 4, Foundation Building, Herston Road, Herston, QLD, 4029, Australia

**Keywords:** Animal model, Arsenic, Environmental exposure, Growth & development, Lung function

## Abstract

**Background:**

Exposure to arsenic via drinking water is a significant environmental issue affecting millions of people around the world. Exposure to arsenic during foetal development has been shown to impair somatic growth and increase the risk of developing chronic respiratory diseases. The aim of this study was to determine if *in utero* exposure to low dose arsenic via drinking water is capable of altering lung growth and postnatal lung mechanics.

**Methods:**

Pregnant C57BL/6 mice were given drinking water containing 0, 10 (current World Health Organisation (WHO) maximum contaminant level) or 100μg/L arsenic from gestational day 8 to birth. Birth outcomes and somatic growth were monitored. Plethysmography and the forced oscillation technique were used to collect measurements of lung volume, lung mechanics, pressure-volume curves and the volume dependence of lung mechanics in male and female offspring at two, four, six and eight weeks of age.

**Results:**

*In utero* exposure to low dose arsenic via drinking water resulted in low birth weight and impaired parenchymal lung mechanics during infancy. Male offspring were more susceptible to the effects of arsenic on growth and lung mechanics than females. All alterations to lung mechanics following *in utero* arsenic exposure were recovered by adulthood.

**Conclusions:**

Exposure to arsenic at the current WHO maximum contaminant level *in utero* impaired somatic growth and the development of the lungs resulting in alterations to lung mechanics during infancy. Deficits in growth and lung development in early life may contribute to the increased susceptibility of developing chronic respiratory disease in arsenic exposed human populations.

## Background

Arsenic is a toxic metalloid that contaminates drinking water through both natural and anthropogenic sources [1]. Millions of people around the world are exposed to arsenic in drinking water at levels above the current World Health Organisation (WHO) maximum contaminant level (MCL) of 10μg/L [2,3]. Several studies have established the link between arsenic exposure and increased lung cancer risk; however, emerging data suggest an important role for arsenic in non-malignant lung disease. Epidemiological data have linked arsenic exposure via drinking water to chronic cough [4], chronic bronchitis [5], bronchiectasis [6] and obstructive lung diseases [7]. Ingestion of arsenic in drinking water has also been associated with respiratory symptoms such as shortness of breath and morning cough [8], and decrements in lung function, including reduced FEV_1_ (forced expiratory volume in 1 s) and reduced forced vital capacity [8-10].

Of particular concern, is the mounting evidence that exposure to arsenic in foetal or early postnatal life has the greatest effect on respiratory health. Exposure to arsenic *in utero*, through maternal drinking water, has been shown to alter the expression of genes in the lungs at birth in rats [11], and result in the development of pulmonary tumours in adult mice [12]. Following a discrete exposure event in Antofagasta, Chile in the 1950-1970s, it was found that exposure to arsenic *in utero* and early postnatal life resulted in a standardised mortality ratios for bronchiectasis in young adults of 46.2 (95% CI, 21.1–87.7) compared to 12.4 (95% CI, 3.3–31.7) if the exposure only occurred postnatally [13]. These data suggest that early life exposure to arsenic can have profound effects on lung health decades after the exposure event.

Exposure to environmental toxins *in utero* may have significant effects on growth and organ development, and have long term implications for disease risk [14]. Arsenic is a known transplacental toxin which is able to cross the placenta and enter foetal circulation at levels equivalent to maternal circulation [15]. In contrast, low levels of arsenic are excreted in the breast milk and breastfeeding is thought to be protective in arsenic exposed areas [16]. In arsenic exposed areas of Bangladesh and West Bengal, exposure to arsenic during pregnancy is associated with infants being born small for gestational age [17] and at a greater risk of developing lower respiratory tract infections in infancy [18]. A mouse study investigating the effects of *in utero* and postnatal exposure to arsenic, at the current WHO MCL of 10μg/L, found that exposed offspring had significant deficits in growth after birth compared with unexposed offspring [19], highlighting the potency of arsenic as an early life toxicant.

Exposure to arsenic during the critical window of development in foetal life may not just restrict growth, but also impair development of the lungs [20]. Foetal growth restriction is associated with worse lung function and greater respiratory morbidity in early childhood [21-23] and adulthood [24,25]. In this study, we determined the effects of *in utero* exposure to low dose arsenic on somatic growth, lung growth and lung mechanics using a mouse model. We hypothesised that *in utero* arsenic exposure would alter the growth and development of the lungs resulting in impairments to postnatal lung mechanics.

## Methods

### Animals and exposure protocol

Time-mated C57BL/6 dams (Animal Resource Centre; Murdoch, Western Australia) were housed at the Telethon Institute for Child Health Research with a 12 h: 12 h light/dark cycle. All experiments were conducted with the approval of the Telethon Institute for Child Health Research Animal Ethics Committee and conformed to the guidelines of the National Health and Medical Research Council of Australia. Dams were given drinking water containing 0 (control), 10 or 100μg/L arsenic in the form of soluble sodium arsenite (NaAs_2_O_3_). This water was provided *ad libitum* from day 8 gestation to delivery (~ day 20 gestation) which ensured that the foetus was exposed to arsenic prior to the development of the lung buds (e9) [26]. Concentrations of arsenic in drinking water were confirmed by inductively coupled plasma-mass spectrometry (ICP-MS; Geotechnical Services, Western Australia). Maternal water consumption, gestation period and litter size were recorded along with somatic growth (body weight and snout-vent length) at birth and the day lung function measurements were taken. Lung function was assessed in male and female offspring at two, four, six and eight weeks of age.

### Animal preparation

Mice were anaesthetised by intraperitoneal injection of a mixture containing xylazine (2mg/mL; Troy Laboratories, New South Wales, Australia) and ketamine (40mg/mL; Troy Laboratories, New South Wales, Australia) at a dose 0.1mL/10g body weight. Mice were tracheotomised and a tracheal cannula inserted (23G stainless steel for two and four week old mice; 1.26 mm outer diameter polyethylene tube for six and eight week old mice) and secured with suture. Mice were ventilated (MiniVent, Harvard Apparatus, Germany) at a tidal volume of 8mL/kg, respiratory rate of 400 breaths per minute and positive end expiratory pressure of 2 cmH_2_O. This elevated respiratory rate was used to suppress spontaneous breathing thus allowing the apnoeic periods required for measurement of lung function without the need for paralysis.

### Thoracic gas volume

Plethysmography was used to measure thoracic gas volume (TGV) as described previously [27]. Briefly, the trachea was occluded at end expiration (transrespiratory pressure, P_rs_ = 0 cmH_2_O) and the intercostal muscles were stimulated with intramuscular electrodes to induce inspiratory efforts. Six 20V pulses of 2-3ms in duration were delivered over a 6s period while recording changes in tracheal pressure and plethysmograph box pressure. TGV was calculated using Boyle’s law after correcting for the impedance and thermal properties of the plethysmograph [27].

### Baseline lung mechanics

Lung mechanics were measured using the forced-oscillation technique as described previously [28]. The forcing function (9 frequencies from 4 – 38 Hz) was generated by a loudspeaker and delivered to the animal via a wave tube during pauses in ventilation. The respiratory system impedance spectrum (Z_rs_) was measured and a 4-parameter model with constant phase tissue impedance was fitted to the data to partition Z_rs_ into components representing the mechanical properties of the airways and parenchyma [29]. This model allowed the calculation of airway resistance (R_aw_) and inertance (I_aw_) and coefficients of tissue damping (G) and elastance (H). The resistance and inertance of the tracheal cannula were subtracted from R_aw_ and I_aw_ respectively. As most of the inertance is contained in the tracheal cannula, values of I_aw_ were insignificant and are not reported.

### Volume dependence of lung mechanics

The volume dependence of lung mechanics was assessed during a slow inflation-deflation (ID) manoeuvre from 0 to 20 cmH_2_O P_rs_. Inspiration was induced by applying a controlled negative pressure to the plethysmograph and expiration was achieved by the slow equilibration of the plethysmograph to atmospheric pressure through a resistor. During the manoeuvre, an oscillatory signal was applied to the lung as described above. The 4-parameter model was fitted to the Z_rs_ using 0.5s data epochs extracted from the signal during the recording period. By monitoring plethysmograph pressure, integrating flow and calculating starting TGV we were also able to construct absolute pressure-volume curves.

### Experimental protocol

To establish a volume history two ID manoeuvres were performed separated with 5 min of regular ventilation. Two measurements of TGV were recorded followed by 6 measurements of baseline lung mechanics at 0 cmH_2_O P_rs_. TGV was recorded again before a final ID manoeuvre (used for analyses).

### Statistical analysis

Maternal and birth outcome means were compared by one-way ANOVA using GraphPad Prism (Version 5.02, GraphPad Software, San Diego, CA USA). Somatic growth, TGV and lung mechanics within each sex were analysed by two-way ANOVA using Sigma Plot with Holm-Sidak post-hoc analysis (Version 11.0, Systat Software, Chicago, IL USA). A p value of less than 0.05 was considered significant.

## Results

### Effects of exposure to arsenic on maternal, birth and growth outcomes

**Figure 1:**
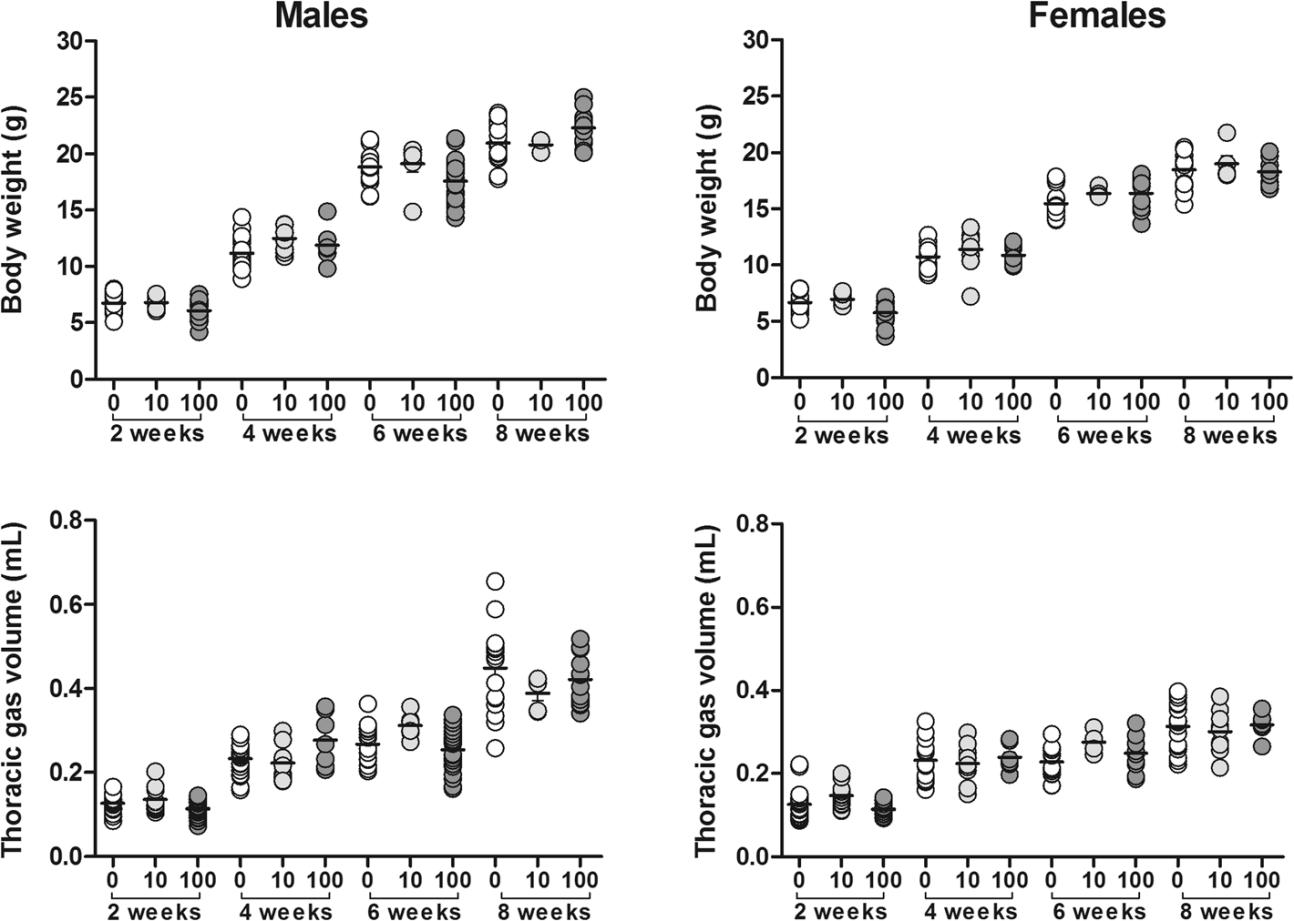
**Body weight and thoracic gas volume in two, four, six and eight week old male and female C57BL/6 offspring exposed*in utero*to drinking water containing 0 (control), 10 or 100μg/L arsenic (mean).** There were no differences in body weight or thoracic gas volume between offspring exposed to arsenic and controls at two, four, six or eight weeks of age.

There was no difference in the amount of water consumed per day between dams exposed to either concentration of arsenic or control water (p = 0.57), nor were there any differences in litter size (p = 0.45) or gestational period (p = 0.32) between arsenic and control water exposed mice (Table 1). At birth, offspring exposed *in utero* to 100μg/L arsenic were significantly smaller in weight (p < 0.001) and length (p < 0.001) than controls, but there were no differences in birth weight or length between offspring exposed to 10μg/L arsenic and controls (p > 0.47) (Table 1). The number of offspring tested from each exposure group at each age is shown in Table 2. There was no effect of *in utero* arsenic exposure on body weight in male (p > 0.58) or female (p > 0.22) offspring at two, four, six or eight weeks of age. Likewise, there was no effect of arsenic on TGV in males (p > 0.30) or females (p > 0.57) at any age (Figure 1).

**Table 1:** Maternal and birth outcomes for C57BL/6J dams exposed to 0 (control), 10 or 100μg/L arsenic via drinking water (mean ± SD)

	**Control**	**10μg/L As**	**100μg/L As**
**Maternal water consumption (mL/day)**	5.11 ± 1.41	4.97 ± 1.36	4.91 ± 1.33
**Gestation period (days)**	20.1 ± 0.92	20.4 ± 0.88	20.0 ± 0.81
**Litter size (pups/dam)**	5.63 ± 1.98	4.79 ± 1.87	5.54 ± 2.23
**Birth weight (g)**	1.34 ± 0.16	1.35 ± 0.19	1.27 ± 0.18 *
**Birth length (mm)**	29.1 ± 1.74	28.8 ± 1.94	28.4 ± 1.90 *

There were no differences in maternal water consumption, gestation period or litter size between control and arsenic exposed mice. Offspring exposed to 100μg/L arsenic were born significantly smaller than control mice in both weight and length (* indicates significantly different to controls).

**Table 2:** Numbers of offspring tested at each age from each exposure group.

**Numbers of offspring tested**	**Control**	**10μg/L As**	**100μg/L As**
**2 weeks - males**	24	11	23
**2 weeks - females**	25	13	15
**4 weeks - males**	22	9	7
**4 weeks - females**	13	9	9
**6 weeks - males**	14	7	24
**6 weeks - females**	14	4	11
**8 weeks - males**	17	5	12
**8 weeks - females**	17	9	12

### Effects of *in utero* exposure to arsenic on early life lung mechanics

**Figure 2:**
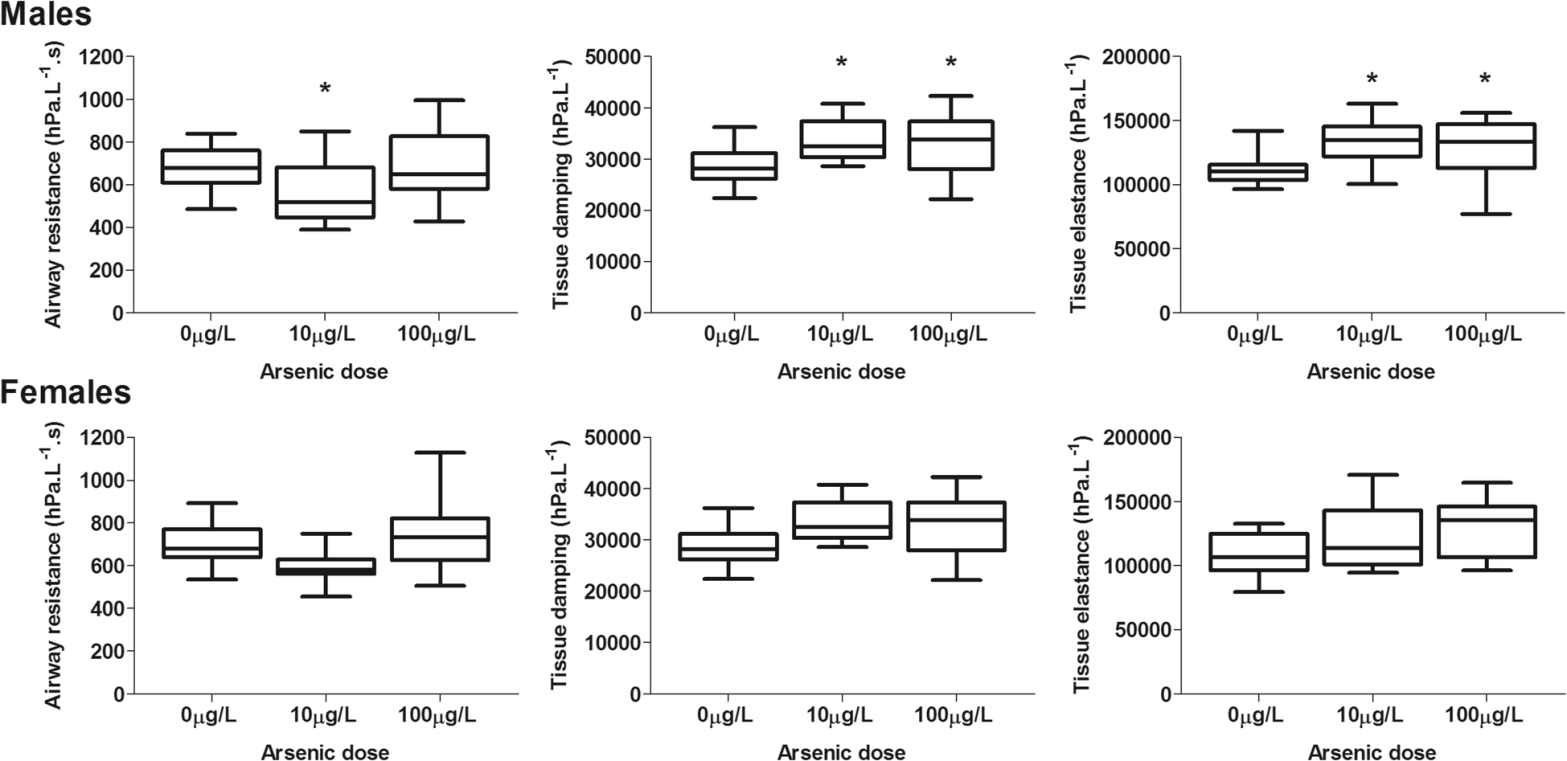
**Airway resistance, tissue damping and tissue elastance at P**_**rs **_**= 0 cmH**_**2**_**0 in two week old male and female C57BL/6 offspring exposed *in utero *to drinking water containing 0 (control), 10 or 100μg/L arsenic (mean ± SD).** Tissue damping and tissue elastance were higher in two week old male offspring exposed to 10 or 100μg/L arsenic compared with controls. There was no effect of arsenic, at either dose, on lung mechanics in two week female offspring (* indicates significantly different to controls).

Two week old male offspring exposed to 10μg/L and 100μg/L arsenic had higher tissue damping (p < 0.001) and tissue elastance (p < 0.001) compared to male controls (Figure 2). Two week old male offspring exposed to 10μg/L arsenic had lower airway resistance (p = 0.03) compared to male controls (Figure 2). There were no differences in airway resistance between two week old male mice exposed to 100μg/L arsenic and male controls (p > 0.42). Although female mice showed similar trends in the data as males, there were no significant effects of arsenic on parenchymal lung mechanics (G, p = 0.31; H, p = 0.64) or airway resistance (R_aw,_ p = 0.14) in two week old female offspring exposed *in utero* to 10 or 100μg/L arsenic via drinking water (Figure 2).

**Figure 3:**
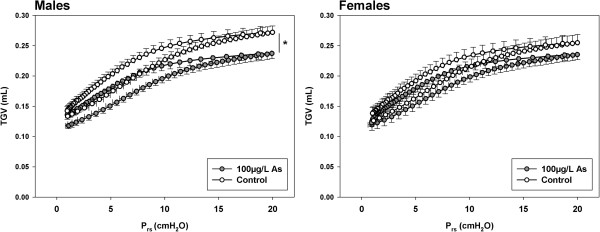
**Pressure-volume curve during inflation to P**_**rs **_**= 20 cmH**_**2**_**O and exhalation to functional residual capacity plotted against TGV in male and female two week old C57BL/6 offspring exposed *in utero *to drinking water containing 0 (control) or 100μg/L arsenic (mean ± SD).** The maximum TGV reached during inflation was lower in male offspring exposed to 100μg/L arsenic compared to male controls. There was no effect of 100μg/L arsenic on maximum TGV in two week female offspring (* indicates significantly different to controls).

The maximum TGV reached during inflation to P_rs_ = 20 cmH_2_0 was significantly lower in two week old male offspring exposed to 100μg/L arsenic (p = 0.02) compared to male controls (Figure 3). Exposure to arsenic at 10μg/L *in utero* had no effect on maximum TGV during inflation in two week old males (p > 0.95 in all cases). There were no significant effects of arsenic, at either dose, on maximum TGV at P_rs_ = 20 cmH_2_0 in two week old female offspring (p > 0.41 in both cases) (Figure 3).

**Figure 4:**
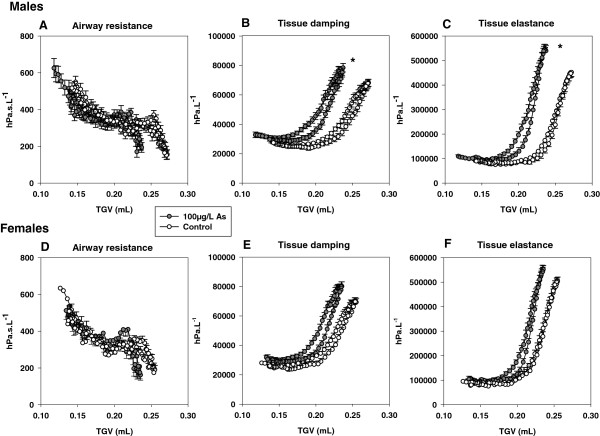
**Volume dependence of airway resistance, tissue damping and tissue elastance during inflation to P**_**rs **_**= 20 cmH**_**2**_**O and exhalation to functional residual capacity in two week old male (A, B, C) and female (D, E, F) offspring exposed *in utero *to drinking water containing 0 (control) or 100μg/L arsenic (mean).** Tissue damping and tissue elastance at P_rs_ = 20 cmH_2_O were significantly higher in male offspring exposed to 100μg/L arsenic compared to male controls. There was no effect of arsenic exposure on tissue mechanics in females and no effect of arsenic on airway resistance in either sex (* indicates significantly different to controls).

Two week old male offspring exposed to 100μg/L arsenic had significantly higher tissue elastance and tissue damping at P_rs_ = 20 cmH_2_0 compared to male controls (H p = 0.02; G p = 0.03, Figure 4). Exposure to arsenic at 10μg/L *in utero* had no effect on tissue elastance or tissue damping at P_rs_ = 20 cmH_2_0 in two week old males (G and H, p > 0.33). There were no differences in airway resistance at P_rs_ = 20 cmH_2_0 between two week old male offspring exposed to arsenic and control water (p > 0.21 in all cases, Figure 3). Exposure to arsenic at 10 or 100μg/L *in utero* had no effect on parenchymal mechanics (G or H, p > 0.54) or airway resistance (R_aw_ p > 0.30, in all cases) at P_rs_ = 20 cmH_2_0 in females at two weeks (Figure 4).

### Effects of *in utero* exposure to on lung mechanics throughout life

**Figure 5:**
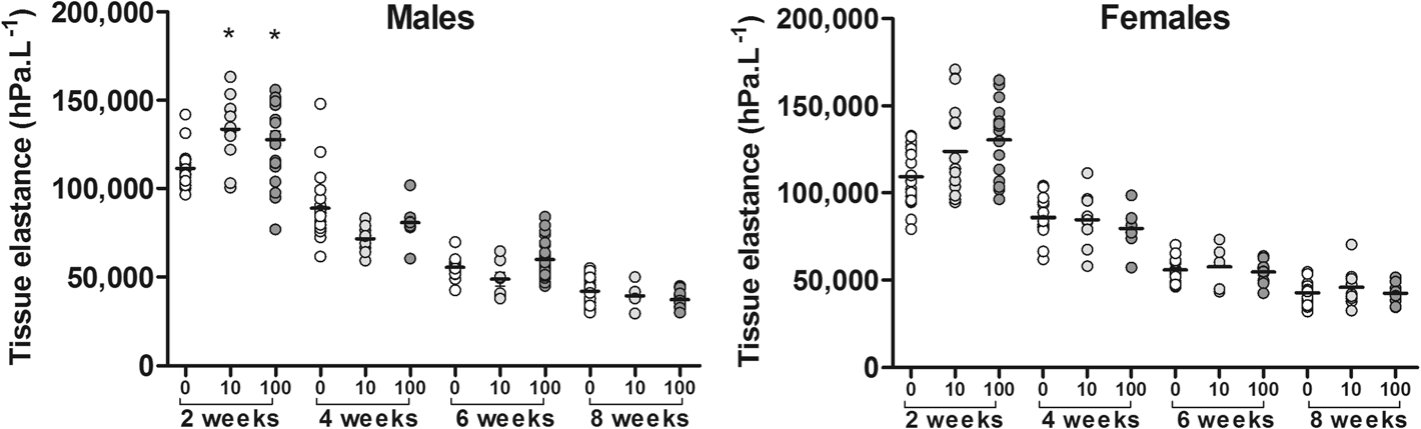
**Tissue elastance at P**_**rs **_**= 0 cmH**_**2**_**0 in two, four, six and eight week old male and female C57BL/6 offspring exposed *in utero *to drinking water containing 0 (control), 10 or 100μg/L arsenic (mean).** Arsenic-induced impairments to lung mechanics in male two week old offspring were resolved at four, six and eight weeks of age (* indicates significantly different to controls).

There were no significant effects of arsenic on tissue damping (p > 0.31) or tissue elastance (p > 0.61, Figure 5) in male or female offspring at 4, 6 or 8 weeks of age. There were also no effects of arsenic on airway resistance (p > 0.34) in either male or female offspring at 4, 6 or 8 weeks of age (data not shown). There were no effects of arsenic maximum TGV at P_rs_ = 20 cmH_2_0 in male or female offspring at 4, 6 or 8 weeks of age compared to controls (p > 0.47 in all cases, data not shown). There were also no effects of arsenic on airway or parenchymal mechanics at P_rs_ = 20 cmH_2_0 in male or female offspring at 4, 6 and 8 weeks of age (p > 0.21 in all cases, data not shown).

## Discussion

In this study a brief exposure of pregnant mice to environmentally low doses of arsenic altered *in utero* growth and lung development of their offspring resulting in deficits in postnatal lung mechanics. This is the first study to show that *in utero* exposure alone to low doses of arsenic via drinking water is capable of impairing postnatal lung function. Exposure to the current WHO MCL of 10μg/L arsenic significantly impaired lung function suggesting that the current MCL may fail to protect against the non-malignant effects of arsenic on the lung. The lung mechanics of male mice were impaired more than those of female mice indicating that males may be more susceptible to the adverse effects of arsenic on the lungs. These deficits in lung mechanics were evident in early life but resolved with age.

Arsenic exposure had a direct effect on *in utero* growth in male offspring exposed to 100μg/L resulting in lower birth weight and length in males compared with controls. There were no differences in the gestational age at birth between offspring exposed to arsenic compared with controls. These mice were therefore small for gestational age, indicating that arsenic exposure *in utero* caused intrauterine growth restriction. Similarly in epidemiological studies, exposure to low dose (<100μg/L) arsenic during pregnancy was linked to low birth weight and size but not to gestational age [17,30]. The mechanism for this is not fully understood but may be a result of arsenic-induced oxidative stress resulting in placental insufficiency [31] or disruption of the endocrine control of glucose homeostasis and cellular growth [32,33]. Being small for gestational age can induce alterations in metabolism, hormonal output and distribution of cardiac output, and have a lifelong impact on the potential for development and survival [34,35]. Foetal growth restriction is associated with increased respiratory morbidity during early childhood [21,22,36] and children who are small for gestational age are more likely to be hospitalised for respiratory tract infections [37,38]. Low birth weight is significantly associated with worse lung function in adulthood and increased mortality from obstructive lung disease [20,24]. There are clear associations between maternal smoking during pregnancy and small for gestational age infants [39], reduced lung function [40] and increased respiratory infections in early childhood [41]. Therefore, arsenic-induced intrauterine growth restriction resulting in low birth weight infants may play a significant role in the childhood morbidity and increased the susceptibility to obstructive lung disease seen in arsenic exposed populations.

Despite the offspring exposed to 100μg/L arsenic being smaller at birth, these mice were not smaller in weight or length at two, four, six and eight weeks of age compared with age-matched controls. In a similar study by Kozul *et al.* mice exposed to 10μg/L arsenic *in utero* had normal birth weight, but did have deficits in body weight during infancy compared with controls [19]. The low birth weight offspring in our study displayed catch-up growth during the first two weeks of life. There is evidence that infants who demonstrate catch-up growth and show early and complete recovery from intrauterine growth restriction are at greatest risk for developing metabolic diseases [42]. Similarly, in low birth weight infants, weight gain during infancy has been shown to be inversely related to lung function in infancy [43], with one study finding that the lowest lung function outcomes were seen in infants of below average birth weight with above average postnatal weight gain [44]. Therefore, despite showing no deficits in body weight later in life, the catch-up growth shown in the first two weeks of life may be related to intrauterine stress and poorer outcomes later in life.

As well as altering the pattern of somatic growth, we have shown that *in utero* exposure to low doses of arsenic (10 and 100μg/L) had direct effects on lung development in males resulting in deficits in lung function two weeks after birth. The abnormalities that we detected were mostly in the parenchymal tissue of the lungs in arsenic exposed offspring. Both tissue elastance and tissue damping were significantly higher in male mice exposed to arsenic compared with male controls, indicative of increased stiffness of the parenchymal lung tissue. When inflated to P_rs_ = 20 cmH_2_O, male offspring exposed to 100μg/L arsenic reached a lower maximum TGV than controls signifying a loss of lung compliance. These data suggest that intrauterine stress caused by arsenic exposure is hindering the development of the distal airways and alveolar tissue in the lungs. Animal models of early life arsenic exposure, that included exposure post-natally, have shown both increased smooth muscle actin in small airways [45], which would increase the resistance of the small peripheral airways (tissue damping), and increased expression of collagen and elastin in the lung parenchyma [46], which would increase tissue stiffness (tissue elastance). As it is well established that sufficient nutrition *in utero* is essential for the healthy development of the lungs [47,48], it is unclear from this study whether the changes in lung mechanics in arsenic exposed mice are a result of suboptimal nutrition during development or a direct effect of arsenic itself.

In our study, male offspring were more susceptible to the harmful effects of *in utero* arsenic exposure on the lung than female offspring. While female mice showed similar trends in deficits in lung mechanics as males, the deficits were not statistically significant. Epidemiological studies also indicate that males may be more susceptible to the health effects of arsenic than females [8,49] which has been attributed to the differences in the way females metabolise arsenic [50]. When inorganic arsenic is ingested, it is absorbed into the blood and transported to the liver where it is reduced and sequentially methylated to monomethylarsonate (MMA) and dimethylarsinate (DMA) [51]. MMA is a highly toxic intermediate product and levels of MMA in the urine correlate strongly with levels of MMA found in the body [52]. DMA is a less toxic end product of arsenic methylation and can also be identified in urine [50]. Females have been shown to metabolise arsenic more efficiently than males, resulting in less MMA and more DMA in the urine [50]. While it is still largely unknown how the effects of arsenic manifest differently in males and females, sex dependent differences in the metabolism of arsenic may have contributed to the difference in susceptibility seen in our study.

We found that *in utero* arsenic exposure alone did not produce a lasting effect on respiratory mechanics, rather the early life deficits recovered with age. This data has two implications from a public health point perspective. Firstly, early life alterations in lung development induced by arsenic exposure may be recovered provided there is access to uncontaminated water. Secondly, we postulate that arsenic-induced impairments lung development may increase the susceptibility to respiratory infections in early life by inhibiting effective clearance of pathogens. This is consistent with the observation that there are increased respiratory infections in children exposed to arsenic-contaminated drinking water *in utero*[18]. Infancy represents a period of high susceptibility to respiratory infections and other harmful environmental exposures. The incidence of respiratory infections leading to bronchitis, pneumonia, or whooping cough in infancy has been shown to reduce adult lung function and increase the risk of mortality from obstructive lung disease [20]. Additionally, animal studies have shown that arsenic compromises the lung’s immune response to viral infections resulting in greater viral load and higher mortality [53]. In humans, early life exposure to arsenic (before age 10) had long term effects on lung function outcomes in adults, with an 11.5% decrease in FEV_1_ and 12.2% decrease in forced vital capacity (FVC) compared to unexposed adults [9]. It is unclear, however, whether the deficits in long term lung function were a result of the direct effects of arsenic on the lung, or an effect of arsenic exacerbating the effects of other respiratory infections or environmental exposures in early life. Arsenic-induced alterations to somatic growth and lung development *in utero* may result in individuals being more susceptible to postnatal insults, and contribute to long term alterations in lung function and disease risk.

## Conclusions

We have shown that the lung is highly susceptible to the adverse effects of arsenic during the *in utero* period of lung development. Exposure to arsenic for a brief period during gestation impaired *in utero* somatic growth and lung development resulting in impaired lung function two weeks after birth (infancy). Alterations to lung function during infancy, a period of high susceptibility to respiratory system insults, may contribute to the increased susceptibility to respiratory infections and diseases in arsenic exposed populations. The deficits following exposure to the current WHO ‘safe’ dose of arsenic highlights the potency of arsenic toxicity and draws attention to the need for ongoing review of regulations regarding safe contaminant levels in community drinking supplies and the particular susceptibility of children to arsenic.

## Abbreviations

WHO: World Health Organisation; MCL: Maximum contaminant level; NaAs_2_O_3_: Sodium arsenite; ICP-MS: Inductively coupled plasma-mass spectroscopy; TGV: Thoracic gas volume; P_rs_: Transrespiratory pressure; Z_rs_: Respiratory system impedance; R_aw_: Airway resistance; I_aw_: Airway intertance; G: Tissue damping; H: Tissue elastance; ID: Inflation-deflation.

## Competing interests

The authors declare that they do not have any competing interests.

## Authors’ contributions

KR was involved in the conception and design of the research question, acquisition, analysis and interpretation of data, drafting and revising the manuscript and approving the final version for publication. AL and PS were involved in the conception and design of the research question, analysis and interpretation of data, revising the manuscript and approving the final version for publication. GZ was involved in the conception and design of the research question, acquisition, analysis and interpretation of data, revising the manuscript and approving the final version for publication.

## Pre-publication history

The pre-publication history for this paper can be accessed here:

http://www.biomedcentral.com/2050-6511/14/13/prepub
